# Have a place to live? – study on the influence of the living environment on the subjective well-being of older adults

**DOI:** 10.1186/s40359-025-03007-1

**Published:** 2025-07-01

**Authors:** Juan Luo, Ziyi Han

**Affiliations:** 1 Pension Insurance and Medical Insurance, School of Management, Shanghai University of Engineering Science, Shanghai, 201600 China; 2 Department of Management, School of Management, Shanghai University of Engineering Science, P.O. Box 201620, Shanghai, China

**Keywords:** Living environment, Older adults, Subjective well-being

## Abstract

**Background:**

Against the backdrop of accelerating global aging, China’s population aged 60 and above has exceeded 18% of the total population, making the quality of life of older adults a focal point of societal concern. As a core factor influencing the quality of home-based elderly care, the living environment encompasses natural, social, material, and spiritual dimensions, directly impacting older adults’ physical and mental health as well as subjective well-being. Existing research has acknowledged the influence of living environments on older adults’ subjective well-being but lacks in-depth analysis of environmental disparities across different regions and cultural contexts.

**Methods:**

Drawing on environmental adaptation theory, socio-emotional selectivity theory, and social support theory, this study utilized 4,298 valid samples from the 2020 China Family Panel Studies (CFPS). A linear regression model was constructed to analyze how differences in living environments—classified into physical, social, and spiritual dimensions—affect older adults’ subjective well-being. Heterogeneity tests were conducted through urban–rural subgroup analyses, and robustness was verified using a winsorization method.

**Results:**

The results show that Indoor air purification, public facility adequacy, surrounding environmental quality, and security all significantly and positively influenced subjective well-being. Neighborhood relationships, community belonging, economic status, and trust in neighbors were positively correlated with subjective well-being. Social support mitigated loneliness, acting as a mediating factor, with a more pronounced effect on older adults living alone.Satisfaction with interpersonal relationships, life confidence, and future expectations positively predicted subjective well-being, while family book collections had no significant impact.Rural older adults were more sensitive to economic security, whereas urban counterparts prioritized quality-of-life factors. Age and educational background showed divergent effects between urban and rural groups.

**Conclusions:**

Disparities in living environments are critical determinants of older adults’ subjective well-being. Enhancing physical infrastructure, strengthening community support, and enriching spiritual life can significantly boost their happiness.

## Introduction

In today’s era, aging has become an irreversible global trend, and older adults have increasingly become a population group that attracts the attention of society as a whole [1]. According to the results of the seventh national population census in 2021, China’s population aged 60 years or above will account for more than 18%. As the largest developing country in the world, how to protect the legitimate rights and interests of older adults and promote the development of the cause of aging is a major issue facing Chinese society, given the growing older adults population base and unbalanced economic development [2]. In the future, the degree of population aging will further increase, and with the development of society and the intensification of population aging, the quality of life of older adults population has received increasing attention. At this stage, most of the elderly choose the home care mode, with institutional care, mutual care and sojourn care accounting for a relatively small proportion. The living environment, as a key factor affecting quality of life, has a profound effect on the quality of home care for older adults and has an important effect on their subjective well-being [3].

The living environment refers to the complexity of various natural, social and cultural conditions or related factors directly related to people’s life and health, including the natural environment, social environment, material environment and spiritual environment [4]. It covers housing conditions, public facilities, environmental health, neighborhood relations, the community atmosphere, social support, mental health, spiritual comfort and other factors and is one of the important factors affecting the quality of human life [5]. The quality of the living environment is directly related to the physical and mental health and quality of life of human beings, so improving the living environment is an important means to improve the quality of life. Older adults are people over the age of 60 years and constitute a special group in society. With increasing age, people are faced with physical function decline, memory loss, vision and hearing decline and other problems but also face psychological loneliness, loss, unease and other emotions. Therefore, paying attention to the mental state of older adults, providing them with a better living environment, improving their subjective well-being, meeting their emotional needs, and improving their quality of life are important issues that society should pay attention to [6]. Happiness is a subjective emotion based on an individual’s judgment of their own quality of life and happiness level. As an important indicator reflecting the quality of life and physical and mental health of older adults, the subjective well-being of older adults is affected by multiple factors of older adults themselves and the outside world, which makes the subjective well-being of older adults vary [7]. As people have increasingly high requirements for quality of life, focusing only on the physical health of older adults is relatively one-sided. As a comprehensive indicator, happiness can effectively measure the overall situation of physical and mental health, emotional status, living standards and other aspects of older adults [8]. Therefore, research on the relationship between the living environment of older adults and their subjective well-being can help us further understand the impact of modernization and population aging on the lives of older adults and provide theoretical and practical support for improving the quality of life and happiness of older adults so that we can explore ways to solve the problem of old-age care from more perspectives.

## Literature review and research hypotheses

### Basic literature review

The influence of the environment on human behavior and health has been widely concerned by different disciplines, and the influence of the environment on older adults is more profound, because the ability of older adults to control the surrounding environment is greatly reduced [9]. Changes in physiological, social and psychological functions limit the adaptability of individuals to stressful environments, so older adults are more susceptible to the influence of external environments. The subjective well-being of older adults refers to the overall evaluation of the quality of life of the individual, a positive, happy and satisfied psychological state, and is one of the important indicators to measure the quality of life of the individual [10]. In international studies on the quality of life of older adults, life satisfaction and happiness are both core indicators to measure people’s well-being, and well-being has an increasing weight in the quality of life, because subjective feelings indirectly or even directly reflect the quality of social services and the satisfaction of older adults with social services. Through the comparative study on the quality of life of older adults in many countries, it is found that in some Nordic countries, the perfect social welfare system and good living environment enable older adults to enjoy high-quality life services, and they have a higher satisfaction with social services and a correspondingly strong subjective well-being [11]. Therefore, this study examined the relationship between living environment and subjective well-being of older adults through nationally representative data. Among them, the living environment is measured by the physical environment and social environment of older adults. The concept of happiness first appeared in psychology, and then was used by sociology, economics, management, sports and other disciplines, and gradually became a research hotspot. At present, the academic circle mainly focuses on the study of subjective well-being, which has gone through three development stages: description and comparison, theoretical construction and measurement improvement, and currently reflects the development trend of deepening, systematization and application [12]. Regarding the subjective well-being of older adults, the academic community initially used a single dimension of gross domestic product (GDP) to measure it, and later developed to measure it by subjective criteria (self-assessment scale) [13].

At present, scholars have conducted extensive and in-depth research on the factors affecting the subjective well-being of older adults, including demographic factors such as gender, age, marriage, economic factors, social support and so on [14]. Domestic and foreign scholars have made more efforts to analyze the factors affecting subjective well-being. Domestic and foreign studies believe that the subjective happiness of older adults is affected by a variety of factors, which can be divided into external factors and internal factors. External factors include economic conditions, self-rated health status, family relations and social network support, while internal factors include personality characteristics and psychological status [15]. Research shows that the two factors of age and education have indirect effects on the well-being of older adults, and the marital status only has indirect and weak effects on the subjective well-being of older adults. Compared with the objective health status, the self-rated health degree of older adults can more effectively predict their subjective well-being. Social support is also closely related to the subjective well-being of older adults, and the more social support older adults receive, the stronger their subjective well-being will be [16]. Economic status is a factor that has a greater direct influence on the subjective well-being of the rural older adults [17]. Many scholars have studied the influence of regional factors on the subjective well-being of older adults [18]. There are huge differences between material and non-material benefits enjoyed by different social status. People will compare their social status, and the higher the social status, the higher the subjective well-being of residents. Nie Jianliang et al. found that living arrangement and living conditions would affect the satisfaction of intergenerational relationship and living conditions of the rural older adults, and further affect their subjective well-being [19]. In general, scholars at home and abroad have analyzed the factors affecting the subjective well-being of older adults from a macro level, but more specific in-depth studies are relatively lacking.

Scholars have been studying the relationship between environment and individual behavior for a long time, especially in the field of older adults. Early studies have found that living environment is closely related to people’s mental health. Communities with harmonious neighbors and frequent organization of various activities have significantly lower incidence of depressive symptoms among elderly people in this community, higher mental health level and stronger subjective well-being [20]. Zhang Jingqiu et al. conducted a study on the living environment satisfaction of the urban older adults in Beijing, and found that the living environment satisfaction of older adults was higher in areas with convenient transportation and complete surrounding supporting facilities. They collected a large amount of data through questionnaires and field interviews with older adults in many urban areas of Beijing, and concluded that factors such as accessibility of public transportation, distance between shopping malls and hospitals have significant effects on older adults’ living satisfaction [21].

Although domestic and foreign scholars have analyzed many factors affecting the subjective well-being of older adults from a macro level, there is still a lack of in-depth research on some specific issues. For example, there are insufficient studies on older adults’ different needs for living environment in different regions and different cultural backgrounds, and how to accurately improve the living environment according to these differences to enhance the subjective well-being of older adults.

### Theoretical analysis and research hypotheses

According to the theory of environmental adaptation, individuals meet their own needs by adapting to the environment to improve their happiness. For older adults, a good living environment can help them better adapt to their living environment and meet their life needs, thus improving their subjective well-being. According to social–emotional choice theory, the goals of older adults’ attention change with increasing age, and they tend to spend time with close social partners and build smaller and more meaningful social networks. According to social support theory, social support is an important factor affecting individuals’ subjective well-being. A good living environment can provide rich social resources and support for older adults, which can help reduce difficulties and pressures in their lives, thus enhancing their subjective well-being [22]. On this basis, conducting an in-depth study on the impact of the gap in the living environment on the subjective well-being of older adults is crucial. This paper further explores the influence mechanism of different living environment factors on the subjective well-being of older adults and how to improve their living environment to enhance their subjective well-being. Based on this,these hypothesis can be proposed.

- H1: The gap of living environment affects the subjective well-being of older adults.
- H2: Physical environment has a positive impact on the subjective well-being of older adults.
- H3: Social environment enhances happiness through the mediating role of alleviating loneliness.
- H4: The mental environment is closely related to the subjective well-being of older adults.

## Data and methods

### Model construction

This paper adopts a linear regression model to investigate the impact of various variables on the subjective well-being of older adults. The model is as follows:

$$\text{SWB}= {\upbeta }_{0}+ {\upbeta }_{1}\text{C}+ {\upbeta }_{2}\text{PYE}+ {\upbeta }_{3}\text{SOE }+{\upbeta }_{4}\text{SPE}+\upmu$$

Among them, SWB is the dependent variable, and C is a series of control variables listed above; PYE is the independent variable for the physical environment, SOE is the independent variable for the social environment, and SPE is the independent variable for the spiritual environment.

### Data source

The data used in this paper are from the 2020 China Family Panel Studies (CFPS) of Peking University, which includes three levels—individual, family and community—and can well reflect changes in population characteristics, such as society, economy and health. The survey objects of the CFPS are households in 25 provinces/municipalities/autonomous regions and all the family members in the sample households, with strong representability, except for special regions or provinces such as Hong Kong, Macao and Taiwan. In this study, older adults aged 60 years and above from the survey data in 2020 were selected as the research objects. After eliminating samples < 60 years old and samples with missing variable information, a valid sample of 4298 cases was finally obtained.

### Setting the core variables

#### Dependent variable

The dependent variable of this paper is the subjective well-being of older adults. The subjective well-being of older adults is graded from 0 to 10 via the questionnaire “where would you place your current overall sense of well—being”, with 0 representing the lowest level of subjective well-being and 10 representing the highest level. This method is objective and is based on an individual’s self-evaluation of quality of life without external interference; thus, it can truly reflect the subjective well-being of the respondents to a greater extent.

#### Independent variable

The independent variable in this paper is the living environment, which is subdivided into three categories: the physical environment, the social environment and the spiritual environment. The physical environment is measured by indoor air purification, public facilities, the surrounding environment and surrounding security. The variables of the social environment include neighborhood relationships, community sentiment, economic status, trust in neighbors, and neighbor help. The spiritual environment is measured by personal relationships, satisfaction with one’s life, confidence in one’s future, and the number of books in one’s household.

#### Control variables

In previous studies, variables were selected mainly from the personal characteristics and family characteristics of older adults, including age, gender, urban and rural areas, educational background and health status at the individual level, and marital status and number of children at the family level. See Table 1.

**Table 1:** Main variables and their explanations

Types of variables	Variable name	Variable description
Dependent variable	Subjective well-being	where would you place your current overall sense of well—being
Independent variable	Physical environment	Air purification Condition of utilities Surrounding conditions Security situation of the surrounding area
	Social environment	Neighborhood relations
		Community affection
		Financial situation
		Trust in neighbors
		Neighbor Help
	Spiritual environment	Popularity situation
		Satisfaction with your life
		How confident you are about your future
		The number of books in the family
Types of variables	Variable name	Variable description
Control variables	Personal characteristic variables	Age
		Gender
		Urban and rural
		Educational background
		Health status
	Family characteristic variables	Marital status
		Number of children

#### Descriptive statistics of the variables

Table 2 lists the variable names, means, standard deviations, minimums, and maximum values for the major variables. As shown in the table, the sample size is 4298, and the subjective well-being of older adults population is relatively high overall, with an average value of 7.789. The distribution of men and women in the sample is relatively balanced. In the self-assessment questionnaire, the overall evaluation of older adults population with respect to external factors such as public facilities, the environment, public security and neighbors’ help is at the middle level. However, elderly people have good feelings about the relationships between them, are satisfied with their lives and have confidence in their future. As shown in Table 2.

**Table 2:** Descriptive statistics

Variables	(1) Observed value	(2) Mean	(3) Standard deviation	(4) Minimum value	(5) Maximum
Subjective well-being Air cleanliness	4298 4298	7.789 0.0686	2.131 0.253	0 0	10 1
Condition of public facilities	4298	2.377	1.035	1	5
Surrounding circumstances	4298	2.361	1.039	1	5
Neighborhood security	4298	2.007	0.915	1	5
Neighborhood collar relationship	4298	1.875	0.850	1	5
Community feelings	4298	1.856	0.867	1	5
Finances Trust in neighbors	4298 4298	1.833 7.127	1.251 2.152	1 0	6 10
Neighbor help situation	4298	1.484	0.861	1	5
Variables	(1) Observed value	(2) Mean	(3) Standard deviation	(4) Minimum value	(5) Maximum
Popularity situation Satisfaction with your life How confident you are about your future	4298 4298 4298	7.397 4.284 4.178	2.054 0.862 0.971	0 1 1	10 5 5
Family book collection Age	4298 4298	1.183 2.105	0.442 1.111	1 1	4 5
Gender	4298	0.520	0.500	0	1
Town and country	4298	0.491	0.500	0	1
Educational background	4298	2.168	1.192	1	7
Health status	4298	3.358	1.257	1	5
Marital status	4298	2.458	1.077	1	5
Number of children	4298	1.914	0.622	1	3

### Empirical results and analysis

#### The influence of the physical environment on the subjective well-being of older adults

The results revealed that indoor air purification, public facilities, the surrounding environment and surrounding security had significant effects on the subjective well-being of older adults. The specific results are shown in Table 3 below. Owing to the use of air purification systems, residences can absorb harmful gases to a certain extent, making elderly people more happy physically and mentally. It also reflects superior living conditions and positively affects the subjective well-being of older adults. The better the public facilities are, the better the surrounding environment, and the safer the surrounding environment is, the greater the subjective well-being of older adults. This is because perfect public facilities and good surrounding environments provide more convenience for older adults, such as convenient transportation, rich shopping choices, high-quality education and medical resources. These convenient conditions help improve the quality of life of older adults, improve their life satisfaction, and thus enhance their subjective well-being. A good surrounding environment usually means good security conditions, reducing the safety risks faced by elderly individuals. A safe environment can make older adults feel more at ease, reduce their anxiety and unease, and thus improve their happiness. Older adults living in such an environment have good physical function and good mood, which directly or indirectly leads to their happiness.

**Table 3:** Influence of the physical environment

	Regression coefficient values	Coefficient standard deviation	t	P >|t|	
Indoor air purification	0.493	0.127	3.90	0.000	F = 35.64
Condition of public facilities	0.125	0.038	3.29	0.001	
Surrounding circumstances	0.120	0.039	3.01	0.003	R^2^ = 0.0312,
Perimeter security	0.197	0.042	4.68	0.000	

#### Influence of the social environment on the subjective well-being of older adults

The results revealed that neighborhood relationships, affection for the neighborhood, economic status and the trust of neighbors had significant effects on the subjective well-being of older adults. Table 4 shows that many factors affect the subjective well-being of older adults and that neighborhood relationships positively affect their subjective well-being. The better the neighborhood relationship is, the happier older adults are. This is because if the neighborhood relationship is good, older adults can interact with their neighbors more frequently, share the trifles in life, and participate in community activities together. These social interactions help alleviate the loneliness of older adults and improve their quality of life. At the same time, interaction with neighbors also contributes to increased self-confidence and social skills and a deeper feeling for the community. Economic status is also an important factor affecting the subjective well-being of older adults and directly affects their living standards. People with good economic status can enjoy more welfare benefits, such as community service and medical security. These factors help improve the life satisfaction of older adults and thus enhance their subjective well-being. The greater the degree to which older adults trust their neighbors in their residential area is, the greater the quality of the people in the community and the friendly atmosphere, which indirectly affects the subjective emotions of individuals.

**Table 4:** Influence of the social environment

	Regression coefficient values	Coefficient standard deviation	t	*P* >|t|	
Neighborhood relations of the community	0.144	0.040	3.61	0.000	F = 136.93, R^2^ = 0.1366,
District feelings Financial situation Trust in neighbors	0.188 0.115 0.312	0.039 0.024 0.015	4.75 4.73 21.30	0.000 0.000 0.000	
Neighbor Help	0.044	0.039	1.13	0.258	

#### Influence of the spiritual environment on the subjective well-being of older adults

The results show that popularity, satisfaction with life and confidence in the future have significant effects on the subjective well-being of older adults. The specific results are shown in Table 5 below. Older adults believe that the better their personal relationship is, the greater their satisfaction with their life, the greater their confidence in their future, and the greater their subjective well-being. A good popularity relationship can provide elderly people with social support. According to social support theory, social support is an important factor affecting an individual’s subjective well-being. When older adults feel lonely or need help, they can obtain comfort or practical help from their friends and neighbors. This type of social support helps improve the self-esteem and self-confidence of older adults, which in turn enhances their subjective well-being. Satisfaction with one’s life is an important indicator of subjective well-being. Older adults generally feel happier and more content if they are satisfied with their lives. This satisfaction can come from many sources, such as family, friends, health status, financial situation, etc. The level of confidence in their future is an important component of mental health in older adults, and older adults generally feel more positive and optimistic if they are confident in their future, which may come from family support, medical security, retirement, etc. At the same time, the degree of confidence in the future is also closely related to the quality of life and mental health of older adults. The above factors directly or indirectly affect the subjective well-being of older adults.

**Table 5:** Effects of the mental environment

	Regression coefficient values	Coefficient standard deviation	t	P >|t|	
The situation of human relations	0.387	0.013	29.21	0.000	F = 585.93,
Satisfaction with your life	0.643	0.037	17.36	0.000	
Level of confidence in your own future	0.345	0.033	10.51	0.000	R^2^ = 0.3531,
Household book holdings	0.093	0.059	1.57	0.117	

#### Heterogeneity analysis

According to the results of the baseline regression, the variables with large average marginal effects among the control variables were further analyzed. Table 6 below shows the heterogeneity analysis of the effects of urban and rural conditions on the living environment and subjective well-being of older adults. As shown in the following table, air purification, surrounding public security, economic status, satisfaction with one’s own life, age, gender and education background are all heterogeneous in urban and rural groups. Older adults living in cities are more likely to pursue quality of life and are more willing to install fresh air systems. Due to the nature of rural areas, compared with those living in urban areas, residential density is lower, and the public security situation is more important in urban areas. Older adults in urban areas have a richer life and more planned income. Therefore, compared with rural areas, economic status has a greater impact on the subjective well-being of older adults, and they pay more attention to their life conditions and have stronger requirements for their life satisfaction.

**Table 6:** Heterogeneity analysis of the impact of the living environment on the subjective well-being of elderly people

	(1)	(2)
	qm2016 Towns	qm2016 Country
air	0.2041^**^	0.0864
	(2.2481)	(0.4538)
ce1	0.0645	0.0568
	(1.4539)	(1.2741)
ce2	0.0677	0.0466
	(1.4209)	(0.9371)
ce3	0.0014	0.0848
	(0.0276)	(1.6682)
ce4	0.0270	0.0174
	(0.5024)	(0.3309)
ce6	0.0885	0.0062
	(1.6253)	(0.1109)
income	0.0540^*^	0.0294
	(1.7877)	(0.8417)
	(1)	(2)
	qm2016 Towns	qm2016 Country
qn10022	0.1262^***^	0.0923^***^
	(5.4537)	(4.0195)
ce5 qm2011	0.0033 0.3088^***^	0.0026 0.3592^***^
	(11.6733)	(14.4408)
qn12012	0.5917^***^	0.5830^***^
	(8.1345)	(8.6886)
qn12016	0.2653^***^	0.3060^***^
	(4.1740)	(5.5595)
fcv age1	0.0572 0.1003^***^	0.0520 0.0942^**^
	(1)	(2)
	qm2016	qm2016
gender	0.1333	0.0009
	(1.8636)	(0.0109)
cfps2020edu	0.0658^**^	0.0501
	(2.1594)	(1.3217)
qp201	0.0534	0.1603
	(1.6649)	(5.0916)
qea0	0.1091	0.0710
	(2.8601)	(1.6849)
chs	0.0520	0.0310
_cons	(0.7921) 1.6013^***^	(0.4341) 1.0859^**^
Number of observations	(3.5556) 2112	(2.5269) 2186
Adjusted R	0.38321088	0.37165746

"*", "* *", and" * * *" respectively indicate significant levels at the 5%, 1%, and 0.1% levels

#### Robustness test

To ensure the authenticity and robustness of the research conclusions, this paper adopts the following shrink-tail method for robustness testing. The robustness test results are shown in Table 7 below. The robustness test results are consistent with the baseline regression results, indicating that the conclusions in this paper are robust.

**Table 7:** Robustness test

	(1)
	qm2016
air	0.1541^*^
	(1.8844)
ce1	0.0053
	(0.1762)
ce2	0.0487
	(1.4447)
ce3 ce4	0.0577 0.0004^***^
	(0.0102)
ce6	0.0370
	(1.0054)
income	0.0527^***^
	(2.4396)
qn10022	0.1093^***^
	(6.9094)
ce5 qm2011	0.0067 0.3433^***^
	(19.8123)
qn12012	0.5916^***^
	(12.9621)
qn12016	0.2730^***^
	(7.0347)
fcv	0.0483
age1	(0.8766) 0.1077^***^
	(4.5566)
gender	0.0822
	(1.5702)
cfps2020edu	0.0675^***^
	(2.8887)
qp201	0.1103
	(4.9820)
qea0	0.0881
	(3.2481)
chs	0.0260
	(0.5710)
_cons	1.2452^***^
Number of observations	(4.2118) 4298
The adjusted R	38656381

"*" and “* * *” respectively indicate significant levels at the 5%, and 0.1% levels

## Summary and discussion

As an important comprehensive index to measure individual quality of life, subjective well-being can reflect the state and psychology of older adults in life to a certain extent. The quality of life of older adults is directly or indirectly related to the living environment, and even determines the quality of various resources available in the process of older adults care. As the Fifth Plenary Session of the 19th Central Committee will actively respond to the aging of the population as a national strategy, the subjective well-being of older adults may receive more attention. With the development of economic and social development, the living environment of older adults has gradually improved, but there is still an undeniable gap. This paper discusses the influence of the gap in the living environment of older adults, including physical environment, social environment and spiritual environment, on their subjective well-being, and draws the following conclusions.

First, the physical environment where older adults live plays an important role in their subjective well-being. The surrounding facilities, environment and public security have a significant positive impact on the subjective well-being of older adults. The perfection of public facilities and public security have a significant impact on the well-being of the urban older adults, which verifies the hypothesis of priority improvement of infrastructure. The effect was more pronounced in older neighborhoods.

Second, the social environment where older adults live also has a positive impact on their subjective well-being, especially the people living in the same community have a significant impact on their individual well-being. Differences in community atmosphere indirectly affect the psychology of older adults, and their satisfaction with life and expectations for life also have an impact on their subjective well-being. Social environment has more influence on the subjective well-being of older adults than physical environment. Neighborhood trust indirectly improves well-being by alleviating loneliness. Especially in older adults living alone, strong neighborhood relationships can compensate for the loss of well-being caused by inadequate physical environment.

Third, among the mental environment factors, people relationship, satisfaction with their life and confidence in their future are closely related, all of which have significant positive effects on the subjective well-being of older adults. Good interpersonal relationships provide rich social support for older adults, help and comfort them when they encounter difficulties or feel lonely, enhance self-esteem and self-confidence, and enhance subjective well-being. High regression coefficient of life satisfaction is an important reflection of subjective well-being, and satisfaction comes from many aspects such as family, friends, health and economy. The regression coefficient of confidence in the future reflects the importance of confidence in the mental health and quality of life of older adults, and confidence may be derived from family support, medical security, pension, etc.

Fourth, the sensitivity of the rural older adults to economic security is higher than that of the urban older adults, which highlights the need to promote economic security and facility improvement simultaneously. Age, gender, educational background also showed different degrees of heterogeneity in urban and rural groups.

On the basis of the above conclusions, this paper proposes countermeasures and suggestions to improve the subjective well-being of older adults and provide them with peace of mind.

First, the government should increase subsidies to improve the community environment. At present, the majority of older adults still choose the home care model, and the internal environment of the residence is crucial for older adults. The government should implement a subsidy policy for older adults housing renovation, provide eligible older adults with air purification systems, barrier-free facilities, emergency calling equipment, etc., and encourage social capital to enter the livable housing market for older adults. Provide housing products suitable for the needs of older adults, and improve the living environment and quality of life of older adults. Increasing investment in public facilities, including parks, libraries, sports facilities, etc., can improve the quality of life of older adults and enhance their happiness. The government promotes the improvement of the green arrangement of public green space, improves the maintenance level, pays attention to the selection of vegetation in front of houses, reduces mosquito bites, pays attention to the greening and beautification of the surrounding environment, reduces noise and pollution, and provides a quiet and comfortable living environment. Urban old communities give priority to promoting the “elevator installation full coverage plan”, focusing on communities with a high proportion of people over 60 years old; Implementation of the “15-min older adults living circle” certification, mandatory new communities with health stations, canteens and barrier-free facilities. We will set up a special fund to adapt rural areas to aging conditions and allocate funds according to the aging rate of the population.

Second, strengthen community property management and build an age-friendly environment. Community property plays an important role in environmental construction, so we should strengthen environmental health management and keep the environment clean. It is also necessary to increase community recreation facilities, improve public green Spaces, open public green Spaces, medical facilities and older adults care facilities. Secondly, increase the utilization rate of residential community squares and parks, and improve the construction of public facilities around communities. The property should also strengthen the surrounding security management, improve security, reduce older adults’ concerns about security issues, set up more monitoring equipment, strengthen patrols, deal with problems in a timely manner, improve older adults’ sense of identity in the residential environment, consider from the perspective of environment and security, in order to improve the quality of life of older adults and enhance their subjective well-being.

Third, call for joint efforts to enhance the sense of gain of older adults. The sense of identity and acquisition of the community will directly or indirectly affect the subjective well-being of older adults. The community should take the lead in strengthening the interaction between neighbors and jointly building a better social environment. Various community activities can be organized to strengthen the interaction and communication between neighbors, so that older adults can feel the warmth and care of the community and find a sense of belonging in the community. Various social activities can be organized, such as senior clubs, volunteer services, etc., to provide social support and communication opportunities for older adults to reduce loneliness; In addition, some public welfare activities can also be provided to allow older adults to actively participate in improving their satisfaction with life. Since individual emotional regulation and mental health also have a huge impact on the subjective well-being of older adults, the community or social institutions can provide psychological counseling and psychological support services to help older adults cope with life pressure and confusion. At the same time, the family members of older adults also need to carry out mental health training and education, so that they can better understand, care for and respect older adults. Gradually realize the full coverage of community psychological service stations, hire professional psychological counselors, provide free depression screening and intervention services, set up “mobile libraries” in rural areas, promote the “rural opera for older adults” project, and use books and local opera performances to enrich the spiritual life of older adults.

Fourth, strengthen community services for older adults and raise public awareness of caring for older adults. The community should provide diversified services for older adults, such as medical care, psychological counseling, etc., to provide convenience and care for older adults. All sectors of society should actively participate in the improvement of the living environment of older adults, provide financial, technical and human support, and jointly create a social environment that cares for older adults. Strengthen publicity and education, improve the public’s attention to and attention to the living environment of older adults, and let more people participate in the improvement of the living environment of older adults. Establish a “time bank” point system, encourage the younger older adults to provide shopping and escort services for older adults, and the points can be exchanged for old-age care services. The community regularly organizes “intergenerational theme activities” every month to enhance the sense of social participation of older adults. The “smart bracelet emergency call system” has been developed to monitor the health data of older adults living alone in real time, train older adults to use community service apps, and integrate functions such as medical registration and housekeeping appointments.

Fifth, pay attention to the differences between urban and rural areas, and formulate differentiated policies according to the different needs of urban and rural older adults. In the city, focus on improving the quality and diversity of public facilities to meet the pursuit of high quality of life for older adults; In rural areas, increase investment in infrastructure construction, improve the economic security level of rural older adults, and narrow the gap between urban and rural areas. For example, providing more employment opportunities and skills training for older adults in rural areas, increasing sources of income, while strengthening rural community construction, improving the living environment, and enhancing the subjective well-being of older adults in rural areas.

The above measures are taken into account mainly at the level of the physical environment and social environment, with the hope that these measures can improve the quality of life of older adults and enhance their subjective well-being. Moreover, it should be noted that older adults’ personal emotions also have a great impact on their subjective well-being. Creating a good social environment is very important for their physical and mental health.

## Data Availability

The dataset of the present study is available upon request from the corresponding author.
